# Diagnostic and prognostic utility of insulinoma-associated protein 1, insulin gene enhancer protein 1, and secretagogin in pheochromocytoma

**DOI:** 10.1590/1806-9282.20250504

**Published:** 2025-10-17

**Authors:** Mehmet Sözen, Gupse Turan, Zeynep Cantürk, Berrin Çetinarslan, Alev Selek, Mesude Tosun, Aziz Hakkı Civriz, Burcu Sevinç, Emre Gezer, Damla Köksalan

**Affiliations:** 1 Kocaeli University, Faculty of Medicine, Department of Endocrinology and Metabolism – Kocaeli, Turkey.; 2 Kocaeli University, Faculty of Medicine, Department of Pathology – Kocaeli, Turkey.; 3 Kocaeli University, Faculty of Medicine, Department of Radiology – Kocaeli, Turkey.; 4 Kocaeli University, Faculty of Medicine, Department of Internal Medicine – Kocaeli, Turkey.

**Keywords:** INSM1, ISL1, Secretagogins, Pheochromocytoma, Immunohistochemistry

## Abstract

**OBJECTIVE:**

The aim of this study was to assess the immunohistochemical expression of second-generation neuroendocrine markers such as insulin gene enhancer protein 1, insulinoma-associated protein 1, and secretagogin in pheochromocytoma and their prognostic value.

**METHODS:**

The study included 30 operated pheochromocytoma patients. The tissue preparations were re-evaluated by two pathologists, and Pheochromocytoma of the Adrenal Gland Scaled Score score and Grading System for Adrenal Pheochromocytoma and Paraganglioma score were given. Four μm-thick paraffin block sections were stained with insulinoma-associated protein 1, insulin gene enhancer protein 1, and secretagogin antibodies to obtain staining intensity score, staining percentage, and H-score.

**RESULTS:**

The mean age at diagnosis was 50.5 (±15.9) years. The most common complaint was high blood pressure. A total of four patients (13.3%) had a nonfunctioning adenoma. The lesions were mostly localized in the right adrenal gland, and the median tumor size was 45.0 (35.0–54.2) mm. The median Ki67 proliferation index was 2.0% (0.9–3.0). According to the Pheochromocytoma of the Adrenal Gland Scaled Score score, nine (30.0%) patients showed the benign clinical behavior (score <4), while according to the Grading System for Adrenal Pheochromocytoma and Paraganglioma score, only four (13.3%) patients were in the well-differentiated-type (0–2 points) group. insulinoma-associated protein 1 and insulin gene enhancer protein 1 were positive in 21 (70%) and 26 (86.7%) of the patients, respectively. However, secretagogin was positive only in six patients (20%). Among the second-generation neuroendocrine immunohistochemical markers, insulin gene enhancer protein 1 had the highest H-score. Correlation analysis showed a negative correlation between insulin gene enhancer protein 1 and tumor Hounsfield unit and a positive correlation between insulinoma-associated protein 1 and Ki67 proliferation index.

**CONCLUSIONS:**

Insulinoma-associated protein 1, insulin gene enhancer protein 1, and secretagogin, which are second-generation neuroendocrine immunohistochemical markers, can be used in the differential diagnosis of pheochromocytoma. Notably, insulinoma-associated protein 1 may also have prognostic significance.

## INTRODUCTION

Pheochromocytomas (PCCs) are rare neuroendocrine tumors (NETs) that originate from chromaffin cells of the adrenal medulla and produce catecholamines. PCCs most commonly present with catecholamine excess symptoms. However, approximately 4–5% of cases are detected incidentally on radiological methods while screening for other causes^1^. Pathologists can recognize PCCs easily when the typical morphology is present; however, it can be challenging in atypical cases. Classically, PCC chromaffin cells are positive for chromogranin A (CgA) and synaptophysin and sustentacular cells are positive for S100. The general opinion is that the most specific neuroendocrine marker is CgA^2^.

PCCs can occur with metastases even a quarter of a century after the initial surgery^3^. Before the recognition that all PCCs should be considered malignant, a number of scoring systems were used to predict the risk of metastasis. However, recent studies have shown that high scores may lead to an overestimation of the risk of metastasis^4^. Therefore, it is an undeniable fact that stronger arguments are needed to predict the risk of metastasis. In recent years, new IHC markers with high diagnostic accuracy have been used for neuroendocrine neoplasms such as insulin gene enhancer protein 1 (ISL1), insulinoma-associated protein 1 (INSM1), and secretagogin (SECG). These molecules are involved in the neuroendocrine cell differentiation steps, and many studies have reported that they are highly sensitive markers in the diagnosis of neuroendocrine neoplasms^5^. In this study, we investigated the immunohistochemical (IHC) expression of ISL1, INSM1, and SECG in PCC tissues in order to define their diagnostic and prognostic value.

## METHODS

All data of PCC patients who were operated on between 2010 and 2021 were retrieved from the hospital archive system, and 30 patients were included in the study. Patients without preparations suitable for staining and patients with mixed tumors were excluded. Radiological re-evaluation was performed by a single radiologist on archived images, via the communication system (Picture Archiving and Communication System [PACS]) (Sectra IDS 7, Sectra, Linköping, Sweden).

The tissue preparations of the study cases were re-evaluated by two pathologists, and Pheochromocytoma of the Adrenal Gland Scaled Score (PASS) score and Grading System for Adrenal Pheochromocytoma and Paraganglioma (GAPP) score were given. A PASS of <4 accurately identified all histologically benign and clinically benign tumors^6^. The total GAPP points were then classified into the following: well-differentiated type (0–2 points), moderately differentiated type (3–6 points), and poorly differentiated type (7–10 points)^7^. After this subclassification, 4-μm-thick paraffin block sections were prepared from all cases and were stained using INSM1 (Cell Marque, 475R-94, 1:25–100), ISL1 (Cell Marque, 431R-14, 1:25–100), and SECG (CellSignaling, CST 14037T, 1:3200) antibodies. The Ki-67 proliferation index was evaluated simultaneously by two pathologists (AHC and GT) using an Olympus BX51 microscope (FN22 10× binocular head). The tumor area was identified using a 4× objective, followed by the identification of hotspot staining areas with a 10× objective. The index was calculated by determining the ratio of tumor cells showing positive nuclear staining in Ki-67 IHC analysis to the total number of tumor cells in five consecutive high-power fields (2.2 mm^2^) observed under a 40× objective (Olympus, 40×, numerical aperture [N.A.] of 0.65, working distance [W.D.] of 0.65 mm)^8^. Staining was performed on a Ventana automatic staining device (Roche Ventana) using Ventana OptiView and UltraView DAB kits. Both staining intensity score (strong=3, moderate=2, weak=1, and absent=0) and staining percentage (0–100) were obtained. The H-score (0–300) was obtained by multiplying the staining percentages and the staining intensity score. Afterward, the H-score expression level was categorized as follows: low (H-score, 0–100), moderate (H-score, 100–200), and high (H-score, 200–300). Ki67 proliferation index and CgA were re-evaluated on previously stained IHC preparations obtained from the archive.

### Statistical analysis

All statistical analyses were performed using SPSS 20.0 (IBM Corp., Armonk, NY, USA). Shapiro-Wilk's test was used to assess the assumption of normality. Continuous variables were presented as mean±standard deviation or (in the case of non-normal distribution) median (interquartile range [IQR]). Categorical variables were summarized as counts and percentages. Associations between continuous variables were determined by Spearman's correlation analysis. Comparisons of continuous variables between groups were carried out using the Mann-Whitney U test and the Kruskal-Wallis test. Dependent-group comparisons were performed by Friedman's two-way analysis of variance. Dunn's test was used for multiple comparisons. A p<0.05 was considered statistically significant.

## RESULTS

The male-to-female ratio in the PCC group was 1:2.75, and the mean age at diagnosis and during the study was 50.5 (±15.9) years and 56.8 (±16.4) years, respectively. A total of eight patients were asymptomatic and were diagnosed incidentally. The most common complaint in symptomatic patients was high blood pressure, of which most was permanent. A total of six patients had a history of malignancy, which comprised prostate cancer (n=1), lip cancer (n=1), colon cancer (n=1), endometrium cancer (n=1), papillary thyroid cancer (n=1), and medullary thyroid cancer (n=1). The patient with medullary thyroid cancer had a REarranged during Transfection (RET) proto-oncogene mutation caused by multiple endocrine neoplasia type 2A (MEN2A) syndrome. A total of 17 patients (56.7%) had norepinephrine-dominant catecholamine elevation, while three patients (10.0%) had epinephrine-dominant catecholamine elevation, and six patients (20.0%) had both catecholamine secretion. We could not detect any increase in the catecholamine secretion of the remaining four patients (13.3%) despite multiple testing attempts.

Genetic analysis was performed in 10 patients (33.3%), of whom seven (23.3%) had no pathogenic mutation. Of the remaining patients, one had SDHD, one had NF, and one had RET mutation.

The lesions were mostly localized to the right adrenal gland and were mostly mixed-characterized lesions with cystic areas, calcifications, and necrosis. The minimum tumor size was 11 mm, while the maximum tumor size was 135 mm. Hounsfield unit (HU) values of all lesions were >10 (min HU:18, max HU:56). All demographic, clinical, and radiological characteristics are given in Table 1.

**Table 1:** Demographic and clinical characteristics.

Variable		Value
Gender (F/M)		22/8
Symptoms		
	Palpitation	18 (60.0%)
	Headache	12 (40.0%)
	Excessive sweating	10 (33.3%)
	Flushing	9 (30.0%)
	Dyspeptic disorder	8 (26.7%)
	Psychiatric state disorder	1 (3.3%)
	Tremor	1 (3.3%)
	Dyspnea	1 (3.3%)
	Hypertension	24 (80%)
Hypertension pattern		
	Sustained	13 (54.2%)
	Paroxysmal	11 (45.8%)
Other diseases		
	Diabetes mellitus	12 (40.0%)
	Cancer	6 (20.0%)
	Coronary artery disease	5 (16.7)
	Cerebrovascular disease	3 (10.0%)
	Hepatitis B	1 (3.3%)
	Asthma	1 (3.3%)
	Dyslipidemia	1 (3.3%)
	Syndromic cases	2 (6.7%)
Biochemical parameters		
	Preoperative urine-free metanephrine (μg/24 h)	239.0 (157.0–1,019.5)
	Preoperative urine-free normetanephrine (μg/24 h)	2,026.0 (1,051.0–4,592.5)
	Postoperative urine-free metanephrine (μg/24 h)	103.0 (53.2–126.0)
	Postoperative urine-free normetanephrine (μg/24 h)	316.5 (250.7–383.0)
Radiological findings		
	CT	7 (23.3%)
	MR	13 (43.3)
	CT+MR	10 (33.4%)
	Tumor size (mm)	45.0 (35.0–54.2)
	Location (right/left)	20 (66.7%)/10 (33.3%)
Structure		
	Solid	11 (36.7%)
	Cystic	6 (20.0%)
	Solid+cystic	13 (43.3%)
	Irregular border	1 (3.3%)
Shape		
	Oval	23 (76.7%)
	Round	7 (23.3%)
	Peripheral tissue invasion	1 (3.3%)
	Peripheral rim	11 (36.7%)
	Calcification	3 (10.0%)
	Necrosis	18 (60.0%)
	Tumor HU	33.06 (±8.75)

CT: computed tomography; HU: Hounsfield unit.

**Figure 1:**
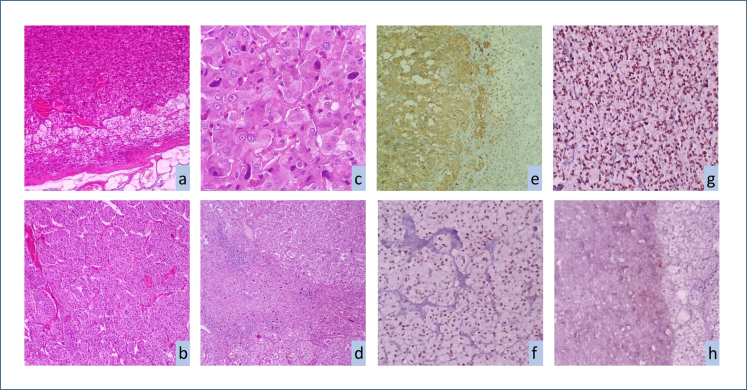
Pathological characteristics of pheochromocytomas. (a) Tumor cells with eosinophilic cytoplasm and oval nuclei compressing the adrenal cortex (HE, 100×). (b) Tumor cells growing in islands (zellballen pattern) (HE, 100×). (c) Nuclear hyperchromasia and nuclear pleomorphism (H&E, 400×). (d) Confluent necrosis (H&E, 200×). (e) Chromogranin-positive tumor tissue (left area). (f) Strongly positive insulinoma-associated protein 1 tumor tissue. (g) Strongly positive insulin gene enhancer protein 1 tumor tissue. (h) Weakly positive secretagogin tumor tissue (left area).

The mean Ki67 proliferation index of all patients was 2.17±1.46. According to the PASS score, most patients were in the potentially aggressive behavior group (score ≥4), while according to the GAPP score, most of them were in the moderately differentiated type (3–6 points) group. All cases were positive for CgA NET marker. INSM1 and ISL1, which are novel second-generation neuroendocrine IHC markers, were positive in 21 (70%) and 26 (86.7%) of the patients, respectively. However, SECG was positive only in six patients (20%) (Table 1). INSM1 and ISL1 staining intensity was mostly weak to moderate, whereas SECG did not show a very large amount of staining (Table 2 and Figure 1).

**Table 2:** Pathological features and immunohistochemical staining intensities.

Ki67 proliferation index (%)		2.0 (0.9–3.0)
<1		7 (23.3%)
1–3		17 (56.7%)
>3		6 (20.0%)
PASS score		4.8 (±2.6)
Benign clinical behavior (score <4)		9 (30.0%)
Potential aggressive behavior (score ≥4)		21 (70.0%)
GAPP score		4.4 (±1.9)
Well-differentiated type (0–2 points)		4 (13.3%)
Moderately differentiated type (3–6 points)		22 (73.4%)
Poorly differentiated type (7–10 points)		4 (13.3%)
CgA staining intensity		
	Negative	0 (0.0%)
	Weak	2 (6.7%)
	Moderate	9 (30.0%)
	Strong	19 (63.3%)
INSM1 staining intensity		
	Negative	9 (30.0%)
	Weak	11 (36.7%)
	Moderate	9 (30.0%)
	Strong	1 (3.3%)
ISL1 staining intensity		
	Negative	4 (13.3%)
	Weak	6 (20.1%)
	Moderate	13 (43.3%)
	Strong	7 (23.3%)
SECG staining intensity		
	Negative	24 (80.0%)
	Weak	5 (16.7%)
	Moderate	0 (0.0%)
	Strong	1 (3.3%)

PASS: Pheochromocytoma of the Adrenal Gland Scaled Score; GAPP: Grading System for Adrenal Pheochromocytoma and Paraganglioma; CgA: chromogranin A; INSM1: insulinoma-associated protein 1; ISL1: insulin gene enhancer protein 1; SECG: secretagogin.

The mean H-score of all cases was positive for CgA at the highest expression level. Among the second-generation neuroendocrine IHC markers, ISL1 had the highest H-score. Their H-score expression levels are summarized in Table 3.

**Table 3:** Expression of traditional and second-generation neuroendocrine immunohistochemical markers.

	CgA	INSM1	ISL1	SECG	p-value		
					CgA vs. INSM1	CgA vs. ISL1	SECG
H-score	300 (200–300)	10 (3.75–80)	120 (20–202.5)	0 (0–0)	0.000	0.075	0.000
Low expression	2 (6.7%)	25 (83.3%)	12 (40.0%)	29 (96.7%)			
Moderate expression	10 (33.3%)	4 (13.3%)	11 (36.7%)	0 (0.0%)			
High expression	18 (60.0%)	1 (3.3%)	7 (23.3%)	1 (3.3%)			

CgA: chromogranin A; INSM1: insulinoma-associated protein 1; ISL1: insulin gene enhancer protein 1; SECG: secretagogin.

Correlation analysis revealed a negative correlation between ISL1 and tumor HU and a positive correlation between INSM1 and Ki67 proliferation index.

None of the patients had metastases at the time of diagnosis, and surgical remission was achieved in all patients. The follow-up period of the patients included in the study was 69 (32.25–106.0) months. In one patient, new liver metastases were detected approximately 24 months after the initial surgery. This patient underwent eight cycles of ^177^Lu treatment. But, the patient died due to progressive disease.

## DISCUSSION

The diagnosis of NETs is based on the histopathological confirmation of neuroendocrine differentiation^9^. Due to the limitations of well-known NET markers in the diagnosis and determination of prognosis, several new markers have emerged, such as INSM1, ISL1, and SECG, which have high sensitivity and specificity levels for diagnosis. These markers have been evaluated in small groups of PCCs in a few NET studies in the literature, but no large PCC-specific studies have been conducted. Therefore, we investigated the clinicopathologic significance of second-generation neuroendocrine IHC markers in PCC patients. In this study, we showed that INSM1 and ISL1 can be used to diagnose PCC, but not SECG. Furthermore, INSM1 expression was positively correlated with higher Ki67 proliferation index levels. Moreover, ISL1 expression showed an inverse correlation with tumor HU on computed tomography (CT) imaging.

INSM1 is a transcription factor involved in the differentiation of embryonic neuroendocrine cells and is expressed in many tumors^10^. Rosenbaum et al. found INSM1 expression in 88.3% of neoplastic NET tissue detected on IHC^11^. In the study by Fujino et al., INSM1 was found to be positive in 71.4% (5/7) of PCC patients and staining intensity rates varied between 5 and 50%^12^. In the study by Möller et al., INSM1 showed strong staining in 72% of patients^13^. The difference in INSM1 staining intensities in the studies may be due to the different clones of the kits used or the decreased antigenic properties of the tissues. Beyond its diagnostic significance, it confirmed that INSM1 is an independent prognostic factor in small-cell lung cancer^14^. Similarly, in pulmonary high-grade neuroendocrine carcinoma patients, overall survival and recurrence-free survival were significantly poorer in the INSM1-positive group^15^. INSM1 was demonstrated by IHC in the majority of cases in this study. In addition, it was found to be associated with Ki67 proliferation index, which has been proposed as a reliable marker to identify cases with a high risk of metastatic spread and poor prognosis in PCC. This result suggests that INSM1 may be a marker that can predict prognosis as well as diagnosis in PCC patients.

ISL1 is a homeobox-gene–related transcription factor involved in pancreatic exocrine and endocrine differentiation and regulates insulin gene expression^16^. Although initial studies suggested that ISL1 immunoreactivity would indicate that the primary site of metastatic NET was the pancreas, subsequent studies demonstrated ISL1 expression in ileal NET, medullary thyroid carcinomas, and PCCs^17–19^. In clinical and preclinical studies, ISL1 showed strong staining intensity in PCC samples^19–21^. In this study, ISL1 immunoreactivity was examined in a large group of PCCs and a high rate of positivity was observed in accordance with the literature. Moreover, it was also similar to CgA in terms of H-score. We found a negative association between ISL1 and tumor HU, but this has never been studied before in the literature. The diagnostic or prognostic significance of this may be improved by further research.

SECG plays a role in cell growth, differentiation, and replication through calcium signaling modulation. SECG immunoreactivity has been demonstrated in many NETs^22,23^. However, in most PCC patients, SECG showed negative or weak immunoreactivity^23^. Juhlin et al. similarly showed negative SECG immunoreactivity in 83.3% (five out of six) of PCC cases^5^. In metastatic NETs of unknown primary, they suggested that PCC or paraganglioma should be investigated in a tumor exhibiting ISL1 (+), INSM1 (+), and SECG (-), according to second-generation neuroendocrine IHC marker results. The present study supports the literature data, and SECG was found to be negative or weakly positive in the majority of cases and could not be associated with prognostic markers.

Our study has several limitations. First, this is a retrospective study, and the small case population may reduce statistical power. Second, we included cases over a 12-year period, so some antigens may have been lost to different degrees or have reduced power. Third, due to technical limitations, the genetic history of all patients is not known, so it is not clear how second-generation neuroendocrine IHC markers work in different genotypes.

## CONCLUSION

Our study demonstrated that INSM1, ISL1, and SECG, which are second-generation neuroendocrine IHC markers, can be used in the differential diagnosis of PCC. Since CgA is sensitive but non-specific, these markers can be used supportively to reveal the nature of a mass. Furthermore, the second-generation IHC NET markers may also have prognostic significance besides their diagnostic role, but further larger studies with a longer follow-up period are needed because metastatic disease may be diagnosed even after 20 years.

## Data Availability

The datasets generated and/or analyzed during the current study are available from the corresponding author upon reasonable request.

## References

[1] Sbardella E, Grossman AB (2020). Pheochromocytoma: an approach to diagnosis. Best Pract Res Clin Endocrinol Metab.

[2] Cheung VKY, Gill AJ, Chou A (2018). Old, new, and emerging immunohistochemical markers in pheochromocytoma and paraganglioma. Endocr Pathol.

[3] Thai E, Gnetti L, Gilli A, Caruana P, Dalla Valle R, Buti S (2015). Very late recurrence of an apparently benign pheochromocytoma. J Cancer Res Ther.

[4] Stenman A, Zedenius J, Juhlin CC (2018). Over-diagnosis of potential malignant behavior in MEN 2A-associated pheochromocytomas using the PASS and GAPP algorithms. Langenbecks Arch Surg.

[5] Juhlin CC, Zedenius J, Höög A (2020). Clinical routine application of the second-generation neuroendocrine markers ISL1, INSM1, and secretagogin in neuroendocrine neoplasia: staining outcomes and potential clues for determining tumor origin. Endocr Pathol.

[6] Thompson LD (2002). Pheochromocytoma of the adrenal gland scaled score (PASS) to separate benign from malignant neoplasms: a clinicopathologic and immunophenotypic study of 100 cases. Am J Surg Pathol.

[7] Kimura N, Takayanagi R, Takizawa N, Itagaki E, Katabami T, Kakoi N (2014). Pathological grading for predicting metastasis in phaeochromocytoma and paraganglioma. Endocr Relat Cancer.

[8] Thompson LDR, Gill AJ, Asa SL, Clifton-Bligh RJ, Krijger RR, Kimura N (2021). Data set for the reporting of pheochromocytoma and paraganglioma: explanations and recommendations of the guidelines from the International Collaboration on Cancer Reporting. Hum Pathol.

[9] Rindi G, Klimstra DS, Abedi-Ardekani B, Asa SL, Bosman FT, Brambilla E (2018). A common classification framework for neuroendocrine neoplasms: an International Agency for Research on Cancer (IARC) and World Health Organization (WHO) expert consensus proposal. Mod Pathol.

[10] Tanigawa M, Nakayama M, Taira T, Hattori S, Mihara Y, Kondo R (2018). Insulinoma-associated protein 1 (INSM1) is a useful marker for pancreatic neuroendocrine tumor. Med Mol Morphol.

[11] Rosenbaum JN, Guo Z, Baus RM, Werner H, Rehrauer WM, Lloyd RV (2015). INSM1: a novel immunohistochemical and molecular marker for neuroendocrine and neuroepithelial neoplasms. Am J Clin Pathol.

[12] Fujino K, Yasufuku K, Kudoh S, Motooka Y, Sato Y, Wakimoto J (2017). INSM1 is the best marker for the diagnosis of neuroendocrine tumors: comparison with CGA, SYP and CD56. Int J Clin Exp Pathol.

[13] Möller K, Uhlig R, Gorbokon N, Dum D, Menz A, Büscheck F (2024). Comparison of INSM1 immunostaining with established neuroendocrine markers synaptophysin and chromogranin A in over 14,000 neuroendocrine and non-neuroendocrine tumors. Mol Cell Endocrinol.

[14] Xu X, Wang G, Duan Y, Huo Z (2022). Prognostic value and non-neuroendocrine role of INSM1 in small cell lung cancer. Pathol Res Pract.

[15] Minami K, Jimbo N, Tanaka Y, Ogawa H, Hokka D, Nishio W (2020). Insulinoma-associated protein 1 is a prognostic biomarker in pulmonary high-grade neuroendocrine carcinoma. J Surg Oncol.

[16] Jensen J (2004). Gene regulatory factors in pancreatic development. Dev Dyn.

[17] Hermann G, Konukiewitz B, Schmitt A, Perren A, Klöppel G (2011). Hormonally defined pancreatic and duodenal neuroendocrine tumors differ in their transcription factor signatures: expression of ISL1, PDX1, NGN3, and CDX2. Virchows Arch.

[18] Graham RP, Shrestha B, Caron BL, Smyrk TC, Grogg KL, Lloyd RV (2013). Islet-1 is a sensitive but not entirely specific marker for pancreatic neuroendocrine neoplasms and their metastases. Am J Surg Pathol.

[19] Agaimy A, Erlenbach-Wünsch K, Konukiewitz B, Schmitt AM, Rieker RJ, Vieth M (2013). ISL1 expression is not restricted to pancreatic well-differentiated neuroendocrine neoplasms, but is also commonly found in well and poorly differentiated neuroendocrine neoplasms of extrapancreatic origin. Mod Pathol.

[20] Hattori Y, Kanamoto N, Kawano K, Iwakura H, Sone M, Miura M (2010). Molecular characterization of tumors from a transgenic mouse adrenal tumor model: comparison with human pheochromocytoma. Int J Oncol.

[21] Dong J, Asa SL, Drucker DJ (1991). Islet cell and extrapancreatic expression of the LIM domain homeobox gene isl-1. Mol Endocrinol.

[22] Dong Y, Li Y, Liu R, Li Y, Zhang H, Liu H (2020). Secretagogin, a marker for neuroendocrine cells, is more sensitive and specific in large cell neuroendocrine carcinoma compared with the markers CD56, CgA, Syn and Napsin A. Oncol Lett.

[23] Lai M, Lü B, Xing X, Xu E, Ren G, Huang Q (2006). Secretagogin, a novel neuroendocrine marker, has a distinct expression pattern from chromogranin A. Virchows Arch.

